# Beyond the Biomarker: Understanding the Diverse Roles of Human Epididymis Protein 4 in the Pathogenesis of Epithelial Ovarian Cancer

**DOI:** 10.3389/fonc.2018.00124

**Published:** 2018-04-24

**Authors:** Nicole E. James, Clinton Chichester, Jennifer R. Ribeiro

**Affiliations:** ^1^Division of Gynecologic Oncology, Program in Women’s Oncology, Department of Obstetrics and Gynecology, Women and Infants Hospital, Providence, RI, United States; ^2^Department of Biomedical and Pharmaceutical Sciences, University of Rhode Island, Kingston, RI, United States

**Keywords:** human epididymis protein 4, epithelial ovarian cancer, tumorigenesis, chemoresistance, metastasis

## Abstract

Human epididymis protein 4 (HE4) is an important clinical biomarker used for the detection of epithelial ovarian cancer (EOC). While much is known about the predictive power of HE4 clinically, less has been reported regarding its molecular role in the progression of EOC. A deeper understanding of HE4’s mechanistic functions may help contribute to the development of novel targeted therapies. Thus far, it has been difficult to recommend HE4 as a therapeutic target owing to the fact that its role in the progression of EOC has not been extensively evaluated. This review summarizes what is collectively known about HE4 signaling and how it functions to promote tumorigenesis, chemoresistance, and metastasis in EOC, with the goal of providing valuable insights that will have the potential to aide in the development of new HE4-targeted therapies.

## Introduction

Approximately 22,280 new cases of epithelial ovarian cancer (EOC) are diagnosed each year, resulting in 14,240 deaths annually in the United States (1). The 5-year survival rate for stage III ovarian cancer is only 39% (1). These dire statistics are due to the fact that the disease is frequently detected at an advanced stage, which drastically impacts overall patient survival. Initially, many patients respond well to first-line therapy that includes cytoreduction surgery and platinum-based treatment. However, many patients experience a chemoresistant recurrence within the first 2 years following treatment (2). Therefore, there is an urgent need for tools to aid in the early diagnosis of ovarian cancer when the disease is fundamentally curable, as well as improved treatment options for later stage disease.

Human epididymis protein 4 (HE4) is a secretory protein that is member of the whey acidic protein domain family, bearing a conserved motif found in a number a protease inhibitors (3). HE4 was initially suggested to be involved in the innate immune defense of multiple epithelia and has also been found to function in epithelial host defense (4). In ovarian tissue, HE4 is highly overexpressed in EOC compared normal tissue (5, 6). Clinically, HE4 has been identified as a novel therapeutic biomarker for EOC and has also proven useful in detection of recurrent disease (7) Serum HE4 level predicts EOC with equal sensitivity to the established biomarker CA125 and is less likely to be elevated in benign disease (5). A multicenter study led by our institution established the FDA-approved Risk of Ovarian Malignancy Algorithm (ROMA), which combines menopausal status and serum levels of both HE4 and CA125 to detect and monitor EOC. ROMA demonstrates improved sensitivity and specificity over the Risk of Malignancy Index that uses CA125 alone as a serum based biomarker (6). Recently, it has been reported that HE4 can be detected in EOC patient urine, indicating the possibility that it may be utilized as a non-invasive biomarker (8).

**Figure 1:**
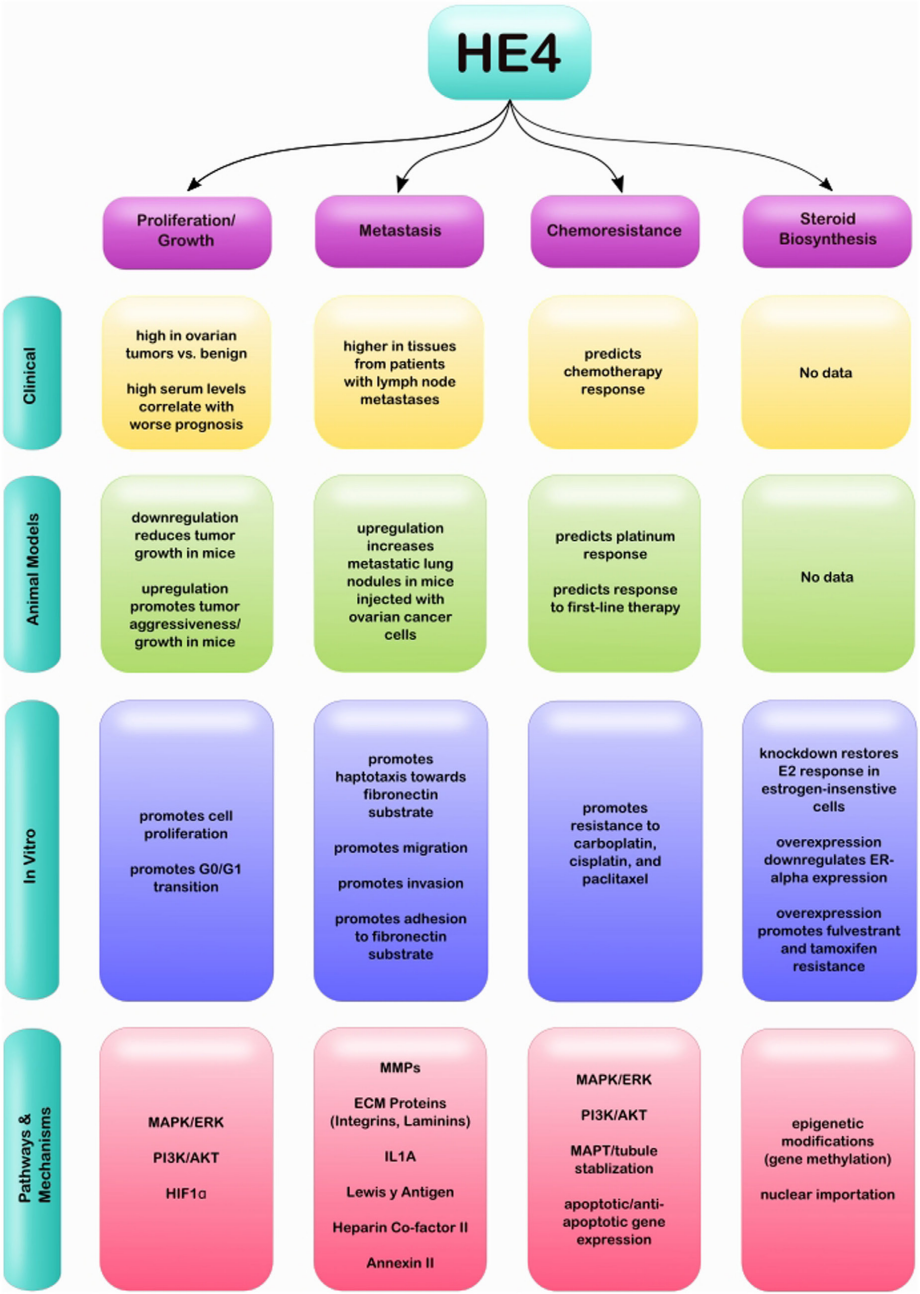
Graphical representation of clinical, *in vivo* and *in vitro* studies completed relating to HE4 and EOC, as well as associated pathways and mechanisms.

While HE4 has been well studied in the clinical setting, less is known regarding its specific molecular and biological roles in EOC. Several studies have investigated its effect on gene expression in EOC cells, as well as on events associated with aggressive disease. This review will summarize HE4’s effect on cell proliferation and tumor growth; invasion, migration, and adhesion; chemoresistance; and steroid biosynthesis (Figure 1). Each section will detail associated pathways and factors that are reported to be involved in these HE4-mediated effects, with the goal of revealing common themes in signaling pathways affected by HE4 and exposing gaps in our knowledge of HE4 molecular and biological functions.

## Cell Proliferation and Tumor Growth

Within the past 5 years, a handful of *in vitro* and *in vivo* studies have begun to examine HE4’s role in proliferation and tumor growth in EOC. A study by Wang et al. examined the role of HE4 in cell proliferation and found that cells treated with recombinant HE4 formed a statistically greater number of colonies compared with control treated cells (9). Furthermore, cells stimulated with recombinant HE4 exhibited greater cell viability compared with respective controls. In another study by Zhu et al. (10), proliferation rate in two different HE4-overexpressing cell lines was significantly higher than in the control cells. Likewise, Zhu et al. (11) and Lee et al. (12) determined that when HE4 was ablated *via* shRNA, cell proliferation decreased accordingly. Kong et al. report conflicting results, stating that HE4 inhibits proliferation in ovarian cells (13); however, no other studies support these claims, necessitating further explanation to understand the implications of their results.

Several *in vitro* studies suggest that HE4 promotes proliferation through its involvement in cell cycle regulation (11). Silencing of HE4 causes G0/G1 cell cycle arrest and blocks the transition from the G1 to the S phase of the cell cycle. Conversely, when cells are stimulated with recombinant HE4, the number of cells in the G2/M phase is increased, while the number of cells in the G0/G1 phase is reduced (9). These results indicate that HE4 may mediate the cell cycle by promoting the G0/G1 transition. In addition, *in vivo* tumorigenicity studies using HE4 knockdown clones revealed a marked inhibition in the growth of ovarian tumors in nude mice (14), while injection of HE4-overexpressing cells led to more aggressive tumor growth and an overall higher tumor volume compared with controls (10, 15). Taken together, results from numerous *in vitro* and *in vivo* studies provide compelling evidence that HE4 plays a role in cell proliferation and the promotion of tumorigenesis. A full list of factors associated with HE4-mediated cell proliferation and tumor growth can be found in Table 1A and is outlined in greater detail below.

**Table 1:** Summary of factors associated with human epididymis protein 4 (HE4) in epithelial ovarian cancer (EOC).

Gene symbol	Description	Association
**(A) Cell proliferation and tumor growth**		
*AKT*	Protein kinase B	Upregulated in overexpressing OVCAR3 HE4 cell lines (10) Decreased in response to HE4 knockdown in OVCAR3 cells (10) Upregulated in HE4 SKOV3 clones compared with vector control (16)

*HIF1*α	Hypoxia-inducible factor-1 alpha	Co-immunoprecipitation with HE4 SKOV3 xenograft tissue (13) Colocalization with HE4 in SKOV3 xenograft tissue (13) Treatment with 2-methoxyestradiol leads to marked decrease in HE4 (13)

*ERK*	Extracellular signal-regulated kinase	Decrease in p-ERK when HE4 was silenced in SKOV3 cells (9) Corresponding decrease and increase in ERK when HE4 was downregulated and overexpressed in OVCAR3 cells (10) Increase in p-ERK in SKOV3 and OVCAR8 cells with recombinant HE4 treatment (16)

*CHUK*	Conserved helix–loop–helix ubiquitous kinase	Upregulation when HE4 was induced in ES-2 cells (8)
*GADD45A*	Growth arrest and DNA-damage-inducible protein GADD45 alpha	
*IL1A*	Interleukin-1 alpha	
*RPS6KA1*	Ribosomal protein S6 kinase alpha 1	
*HSPA1B*	Heat shock 70 kDa protein 1B,	
*DUSP1*	Dual specificity protein phosphatase 1	
*JUND*	Transcription factor JunD	

*EGF/EGFR*	Epidermal growth factor/epidermal growth factor receptor	Co-immunoprecipitation with HE4 SKOV3 xenograft tissue (13) Colocalization with HE4 in SKOV3 xenograft tissue (13) HE4 overexpressed in OVCAR8 cells when stimulated with recombinant protein (13) HE4 increased when inhibited by IRESSA (13)

*VEGF*	Vascular endothelial growth factor	HE4 overexpressed in OVCAR8 cells when stimulated with recombinant protein (13)
*INS*	Insulin	

**(B) Invasion, migration, and adhesion**		
*MMP-9*	Matrix metallopeptidase 9	Downregulated when HE4 is silenced in ovarian cell lines (9)
*MMP-2*	Matrix metallopeptidase 9	
*CTSB*	Cathepsin B	

*IL1A*	Interleukin-1 alpha	Microarray results reveal correlation with HE4 levels (8, 71) One microarray reports an inverse correlation with HE4 (16)

*ITGβ5*	Integrin β5	Differentially regulated by HE4 in ES-2 and CaOV3 cells (8) Correlation with HE4 in paraffin embedded ovarian human tissue (8)

*SDC1*	Syndecan 1	Differentially regulated in response to HE4 (71)
*COL1A1*	Collagen type 1 alpha 1	
*DAG1*	Dystroglycan 1	

*LAMB3*	Laminin-β3	Increased expression when stimulated with recombinant HE4 in OVCAR8 cells (57)
*LAMC2*	Laminin-γ2	
*GREM1*	Gremlin 1	
*TNC*	Tenascin C	
*SERPIND2*	Serine peptidase inhibitor member 2	

*LAMA3*	Laminin 332	Increased in response to HE4 stimulation of *LAMB3* and *LAMC2* (57)
	Lewis y antigen	Colocalized with HE4 in human ovarian tissue (98) Immunohistochemistry stained found correlative staining with HE4 (98) Overexpression promoted HE4-mediated invasion and metastasis in *in vitro* cell lines (99) Knockdown promoted a decrease in invasion and metastatic properties of HE4 (99)

*SERPIN D1*	Heparin cofactor II	Upregulated in HE4-overexpressing clones and downregulated in knockout in *in vitro* lines (71) Spearman analysis revealed positive correlation with HE4 in human EOC tissue immunohistochemistry staining (71) Poor patient prognosis when levels upregulated in combination with HE4 (71)

*ANXA2*	Annexin II	Mass spectrometry and co-immunoprecipitation identify as strong interacting partner of HE4 (58) Gene levels co-dependent with HE4 (58) Higher along with HE4 in EOC patients with lymph node metastasis than those without (58)

*LAMB2*	Laminin subunit beta-2	Gene levels decreased in presence of HE4 knockdown cell line (58)
*MKN2*	MAP kinase-interacting serine/threonine-protein kinase 2	

**(C) Chemoresistance**		
*EGR1*	Early growth response protein 1	Suppressed in overexpressing HE4 clones (16)

*p38*	p38 mitogen-activated protein kinase	Activated in NV cells treated with cisplatin and suppressed in overexpressing HE4 clones (16)

*BCL2*	B-cell lymphoma 2	Increased in response to recombinant HE4 *in vitro* (7)

*BAX*	bcl-2-like protein 4	Decreased in response to recombinant HE4 *in vitro* (7)

*MAPT*	Microtubule-associated protein tau	Upregulated in HE4-overexpressing clones (16)
*SEPT3*	Septin 3	

*TUBB*	β-Tubulin	Increased in response to recombinant HE4 *in vitro* (16)

*ERK*	Extracellular signal-regulated kinase	Knockdown with HE4 lead to a reduction in cell growth and resensitization to cisplatin and paclitaxel (10)
*AKT*	Protein kinase B	

**(D) Steroid biosynthesis**		
*FOXA2*	Forkhead box protein A2	Differentially expressed in -overexpressing clones and knockouts (71)

*SQLE*	Squalene monooygenase	Differentially expressed in HE4-overexpressing clones and knockouts (71)
*DHCR7*	Dehydrocholesterol reductase	
*NSDHL*	Sterol-4-alpha-carboxylate-3 dehydrogenase	

*5-MC*	5-Methylcytosine	Downregulated in HE4-overexpressing clones compared with wild-type SKOV3 cells and null vector (131)

*ESR1*	Estrogen/estrogen receptor	Abolished in HE4-overexpressing clones (131) When stimulated in H08910 cells HE4 increased in gene and protein levels. Effect not observed in SKOV3 cells (131) Increase expression in HE4 SKOV3 knockdown (131)

### Associated Pathways and Factors—Cell Proliferation and Tumor Growth

Human epididymis protein 4 has been connected to several oncogenic signaling cascades that play key roles in ovarian cancer progression, including the PI3K/AKT pathway, HIF1α, and ERK/mitogen-activated protein kinase (MAPK) signaling. Evidence of HE4’s effect on activation of each of these pathways is discussed below.

#### Protein Kinase B Signaling

AKT has been established as a strong promoter of tumorigenesis, and the PI3K/AKT pathway is one of the most commonly hyperactivated pathways in many types of human cancers (16). Its diverse signaling regulates proliferation, growth, survival, motility, angiogenesis, and glucose metabolism (17). HE4-overexpressing OVCAR3 ovarian cancer cells were found to have a marked increase in activation of protein kinase B (AKT) compared with control cells, while HE4 knockdown in OVCAR3 cells reduced AKT activation (12). Moreover, it was found that HE4-overexpressing SKOV3 clones had naturally higher gene levels of *AKT3* compared with the null-vector control (18), bolstering the claim that HE4 affects the PI3K/AKT pathway.

#### Hypoxia-Inducible Factor-1 Alpha (HIF1α)

Adaptation of malignant cells to hypoxic conditions is a key step in the promotion of tumorigenesis and angiogenesis (19–21), a process that is regulated by the transcription factor HIF1α. Co-immunoprecipitation revealed an interaction between HIF1α and HE4 in HE4-overexpressing SKOV3 xenografts. There was also strong colocalization of HE4 and HIF1α in SKOV3 ovarian xenograft tissue. In addition, when SKOV3 cells were treated with HIF1α siRNA or 2-methoxyestradiol (a HIF1α inhibitor), there was a marked decrease in HE4 protein levels (15). It is important to note that 2-methoxyestradiol is not a specific HIF1α inhibitor as it primarily causes the depolymerization of microtubules, which in turn prevents HIF1α expression (22). Thus, the specificity of the effect of HIF1α inhibition on HE4 levels may require further investigation. Although the exact mechanism and significance of the HE4-HIF1α interaction is not understood, this evidence suggests that HE4 could play a role in regulating HIF1α functions in angiogenesis.

#### MAPK Signaling

The MAPK pathway is composed of a family of conserved kinases that mediate essential cellular processes such as migration, growth, proliferation, differentiation, and apoptosis (23). The extracellular signal-regulated kinase (ERK) pathway is the best characterized of all MAPK pathways and is deregulated in approximately one-third of all cancers. Several studies have shown activation of ERK in response to HE4 treatment or overexpression, or suppression of ERK phosphorylation in response to HE4 knockdown (11, 12, 18). Using microarray analysis, Zhu et al. determined that seven genes involved in the MAPK pathway (CHUK, GADD45A, IL1A, RPS6KA1, HSPA1B, DUSP1, and JUND) were differentially regulated in response to HE4 overexpression in ES-2 cells (10).

Activation of the MAPK/ERK pathway occurs through EGF binding of its membrane bound receptor, EGFR (24). Using co-immunoprecipitation studies in SKOV3 cells, Moore et al. found that HE4 interacts with EGFR, with a greater degree of immunoprecipitation seen in HE4-overexpressing clones than wild-type cells (15). Furthermore, ovarian xenograft tissue showed colocalization of HE4 and EGFR. In addition, when SKOV3 and OVCAR8 cells were stimulated with growth factors EGF, VEGF, and Insulin, nuclear localization of HE4 was significantly increased. Finally, when EGF was repressed by the small molecule inhibitor Iressa, relative intensity of HE4 staining was decreased in ovarian cancer cell lines. Collectively, these results provide several layers of evidence that HE4 is tied to growth factor signaling and the MAPK/ERK pathway, although further research is needed to elucidate the precise mechanisms involved.

### HE4’s Role in Proliferation in Other Cancers

Human epididymis protein 4 has been investigated as a putative biomarker in endometrial (25–39), lung (40–52), breast (53, 54), pancreatic (55, 56), and gastric cancer (57). While the majority of these studies examine the value of HE4 as a clinical biomarker for detecting and monitoring disease, one study investigated the molecular mechanisms of HE4 in pancreatic and endometrial cancer. Lu et al. stimulated both pancreatic and endometrial cancer cell lines with recombinant HE4 and found that cell viability, cell growth, and DNA synthesis was increased prominently in both cancer types (56). They also report that HE4 upregulates gene expression of proliferating cell nuclear antigen (PCNA) and downregulates p21 in both cancer cell lines in a dose dependent manner. PCNA, which is expressed in the late G1/S phase of the cell cycle, is required for DNA repair, replication, cell proliferation, and cell cycle progression (58), while p21 is an important effector of tumor suppressor pathways by promoting cell cycle arrest. Specifically, p21 is able to facilitate p53-dependent G1 growth arrest (59). Therefore, results from this study highlight HE4’s role in proliferation in both pancreatic and endometrial cancer and lend support to similar evidence from studies published on EOC.

## Invasion, Migration, and Adhesion

Several studies have associated HE4 with metastatic properties, including invasion, migration, and adhesion of ovarian cancer cells. Lu et al. found that adhesion to a fibronectin substrate was twofold greater in SKOV3 cells overexpressing HE4 than in mock cells. In addition, a transwell migration assay demonstrated that the HE4-overexpressing clones had a 1.8-fold greater migration capacity than mock transfected cells. By contrast, immunofluorescence analysis showed that HE4 knockout clones displayed inhibited cell-spreading ability in a statistically significant fashion compared with respective controls. Furthermore, cell invasion, proliferation, and migration were significantly decreased in these clones (14). In agreement with this study, Ribeiro et al. also found that OVCAR8 ovarian cells treated with recombinant HE4 exhibited 2.07-fold greater invasion capacity and 1.29-fold greater adhesion to a fibronectin matrix compared with untreated controls. Interestingly, there was no change in adhesion to collagen I, IV, laminin I, and fibrinogen matrices, suggesting that HE4 has a specific effect on fibronectin adhesion. Haptotaxis toward a fibronectin substrate also was increased in the ovarian cancer cells treated with recombinant HE4 by 1.72-fold (60).

Zhu et al. used wound healing and transwell invasion assays to show that HE4-overexpressing ES-2 and CaOV3 cells possess enhanced cell migration and invasion capacities. In addition, *in vivo* tail vein injection of HE4-overexpressing ES-2 cells into nude mice resulted in significantly more metastatic lung nodules than mock transfected cells (10). Using the same ovarian cancer cell lines, Zhuang et al. report the importance of HE4 interaction with annexin II (ANXA2) to promote invasion and migration *in vitro* and metastasis *in vivo* (61). Finally, Zou et al. found that knockdown of HE4 in SKOV3.ip1 cells inhibited migration and invasion (62). Taken together, these studies strongly suggest that HE4 plays a prominent role in the promotion of ovarian cancer metastasis. A full list of factors associated with HE4-mediated invasion, migration, and adhesion can be found in Table 1B and is outlined in greater detail below.

### Associated Pathways and Factors—Invasion, Migration, and Adhesion

Human epididymis protein 4 appears to interact with numerous molecular pathways that promote metastasis in ovarian cancer. However, it is still not entirely known how HE4 affects signaling pathways and gene expression signatures to promote invasion, migration, and adhesion of ovarian cancer cells. Following is a summary of HE4-mediated molecular pathways that are involved in metastatic events in EOC.

#### Matrix Metalloproteinases (MMPs)

Human epididymis protein 4 has been associated with MMPs MMP-9 and MMP-2, and Cathepsin B. MMPs are a family of zinc-dependent endopeptidases that are vital for the remodeling of the extracellular matrix (63). They are expressed in almost all types of cancers and are responsible for stimulating angiogenesis, tumor growth, and metastasis (64, 65). Cathepsin B is a lysosomal cysteine protease that has been linked to cancer progression (66), specifically in signaling pathways related to angiogenesis (67). In addition, it can promote MMP activity by degrading MMP inhibitors (68). Interestingly, silencing of HE4 in ovarian cancer cells led to a decrease in protein levels of MMP-9, MMP-2, and Cathepsin B, suggesting these factors may be involved in HE4-mediated tumor promoting effects (11).

#### Interleukin-1 Alpha (IL1A)

Interleukin-1 alpha is a pro-inflammatory cytokine that is involved in angiogenesis and metastasis. ILIA can directly stimulate the synthesis of VEGF (69) and fibroblastic pro matrix metallic proteinase I (70, 71). IL1A causes resistance to EGFR inhibitors in both colon and head and neck cancers (72, 73). IL1A was also found to be differentially expressed in three separate microarray studies involving HE4. In two microarrays, IL1A levels positively associated with HE4 levels (10, 74), while in one study their levels were inversely associated (18). While there may be some ambiguity as to how HE4 and IL1A are mechanistically linked, the consistent connection between IL1A with HE4 merits further investigation.

#### Extracellular Matrix Proteins

Integrins are a family of transmembrane proteins that are vital to ECM adhesion and play important roles in wound healing as well as the pathogenesis of cancer (75–77). Integrin β5 (ITGβ5) gene expression was differentially regulated by HE4 in ES-2 and CaOV3 cells, which was confirmed by positive correlation of ITGB5 and HE4 staining in paraffin embedded ovarian tissue samples (10). This finding suggests that integrin signaling is one mechanism by which HE4 can promote increased adhesion of ovarian cancer cells. However, further research is needed to clarify the mechanisms involved.

In addition to ITGβ5, three other genes related to ECM modeling—syndecan 1 (SDC1), collagen type 1 alpha 1 (COL1A1), and dystroglycan 1 (DAG1)—were more highly expressed in cells overexpressing HE4 and were downregulated in cells with HE4 knockdown (10). SDC1, also known as CD138, is an essential cell surface adhesion molecule that is responsible for maintaining cell morphology and interactions within the surrounding microenvironment (78). Loss of SCD1 in cancer cells is associated with reduced ECM adhesion and enhanced invasion and cell motility (79). Another ECM gene found to be affected by HE4 expression levels, COL1A1, is a crucial component of the ECM as it supports cartilage, bone, and tendon tissues in the body and also functions to maintain the rigidity and elasticity of tissues (80, 81). COL1A1 plays an important role in cancer, since tumor cells that express COL1A1 are able to dissociate from their surrounding stromal components, which is essential for tumor growth (81). The final ECM gene found to be affected by HE4 is DAG1, which is a cell adhesion molecule that plays a key role basement membrane assembly (82), muscle integrity (83), and the maintenance of basolateral cell adhesion in numerous epithelial tissues (84). Loss of DAG1 is associated with cancer progression (85). Taken together, these results show that HE4 is strongly interconnected with ECM related proteins, specifically those involved in the ITGβ5 signaling pathway.

Our lab has also determined that HE4 regulates several components of the extracellular matrix (60). We performed microarray analyses comparing untreated OVCAR8 wild-type cells to recombinant HE4 treated cells, and OVCAR8 cells overexpressing HE4 to null-vector control cells. Serpin peptidase inhibitor, member 2 (SERPINB2), gremlin 1 (GREM1), laminin-β3 (LAMB3), laminin-γ2 (LAMC2), fibroblast growth factor 5 (FGF5), and tenascin C (TNC) were all found to be significantly upregulated upon treatment with recombinant HE4. These genes encode for extracellular matrix proteins that promote cell migration and adhesion (60). Specifically, we found that HE4 upregulates LAMC2 and LAMB3 proteins in a time-dependent manner, and this increase of both factors in turn leads to an increase in laminin-332 levels (60). Laminin-332, a heterotrimer composed of LAMC2, LAMB3, and LAMA2, is an important component of the basement membrane in epithelial tissue. Abnormal increases in its levels have been shown to promote increased invasion in cancers (86). Further evidence suggested involvement of the FAK pathway in these events. In addition, activation of matriptase, a serine protease responsible for cleaving laminin-332 in its β chain and regulating its effects on metastatic properties, increased upon *in vitro* exposure to recombinant HE4 (60). This study provides compelling evidence that HE4 is involved in basement membrane invasion and adhesion.

#### Lewis y Antigen

Human epididymis protein 4 undergoes glycosylation before it is secreted by ovarian cells (87), prompting Zhuang et al. to examine the relationship between HE4 glycosylation status and metastatic properties. Lewis y antigen is a glycosyl antigen that is overexpressed in ovarian cancer and has been associated with chemoresistance and poor prognosis (88–97). They determined that Lewis y antigen was present in HE4 from benign and malignant ovarian tissues, *in vitro* cancer cells, and culture medium. HE4 from ovarian cancer samples contained higher levels of Lewis y antigen than HE4 from benign tissues, and their expression co-localized in ovarian cancer tissue (98). Furthermore, when Lewis y antigen was over expressed, it promoted HE4-mediated invasion and metastasis in ovarian cancer cell lines. Conversely, when Lewis y antigen was blocked, the invasive and metastatic properties of HE4 were significantly decreased (99). Interestingly, overexpression of Lewis y antigen increased tyrosine phosphorylation of EGFR and HER/neu, which promoted cell proliferation through the PI3K/Akt and Raf/MEK/MAPK pathways (100). Thus, it appears that Lewis y antigen and HE4 affect similar signaling pathways that promote tumor growth and malignancy (101). Taken together, these results show that Lewis y antigen could be a potential therapeutic target to decrease HE4 function in the treatment of EOC.

#### Heparin Cofactor II (HCII)

SERPIND1 encodes for the protein HCII, which is a serum glycoprotein and protease inhibitor (102). A study in non-small cell lung cancer (NSCLC) showed that HCII promotes cell motility, invasion, and filopodium dynamics through the PI3K/AKT pathway. High HCII expression in NSCLC tissue correlated to an increased recurrence rate and shorter overall survival (103). Furthermore, its levels were upregulated in metastatic brain cell lines compared with non-metastatic parental lines, suggesting an involvement of SERPIND1 in metastatic functions (104). Results from a microarray study by Zhu et al. showed that SERPIND1 was upregulated in HE4-overexpressing cells and conversely downregulated in HE4 knockdown cells. These results were validated *via* qPCR and immunohistochemistry. In addition, they found that 37/50 ovarian cancer samples showed positive expression of both SERPIND1 and HE4, and Spearman correlation analysis confirmed that HE4 and SERPIND1 were positively correlated. Finally, Kaplan–Meier analysis revealed that patients with high levels of HE4 and SERPIND1 had a worse prognosis (74). While these data strongly suggest a connection between HE4 and SERPIND1, which may be related to their roles in promoting ovarian cancer metastasis, further study of the association between these two proteins is required.

#### Annexin II

Annexin II is a calcium-dependent, phospholipid-binding protein that is overexpressed in a variety of cancers and is involved in angiogenesis, proliferation, apoptosis, cell migration, invasion, and adhesion (105). High levels of Annexin II activate MAPK signaling, which in turn promotes tumor proliferation (106), invasion (107), and metastasis (108). Zhuang et al. employed mass spectrometry and co-immunoprecipitation to identify Annexin II (ANXA2) as a strong HE4 interacting partner (61). This binding promoted invasion and metastasis in ES-2 and CaOV3 ovarian cancer cells. *HE4* and *ANXA2* gene expression levels were found to be co-dependent, and examination of EOC tissue revealed that both HE4 and Annexin II levels were increased in malignant phenotypes compared with benign and normal ovarian tissues. Both proteins were also more highly expressed in tissues from patients with lymph node metastases than those without. Downregulation of HE4 was found to decrease expression of MKNK2 (MAP kinase-interacting serine/threonine-protein kinase 2) and LAMB2 (laminin, beta-2), two factors associated with MAPK and focal adhesion signaling pathways. When HE4 protein was supplemented, this effect was reversed. Collectively, these results show that HE4 interaction with Annexin II to activate MAPK and focal adhesion signaling is one mechanism by which HE4 may promote ovarian cancer metastasis.

## Chemoresistance

Several studies show that HE4 is associated with chemoresistance clinically. The addition of HE4 serum levels in the ROMA score better predicts platinum resistance in patients than CA125 alone (15). Angioli et al. found that HE4 was able to predict chemotherapy response in EOC patients undergoing first-line therapy (109). In addition, higher levels of serum HE4 are reported in women who are resistant to first-line chemotherapy (110). Finally, higher HE4 levels inversely correlate with clinical outcome (111), optimal cytoreduction (112), progression free survival (113), and overall survival (15, 113). While the mechanism underlying HE4’s contribution to chemoresistance has not been established fully, a few studies have begun to delineate HE4’s role in this process. A full list of factors associated with HE4-mediated chemoresistance can be found in Table 1C and is outlined in detail below.

### Associated Pathways and Factors—Chemoresistance

#### Antiapoptotic Gene Expression

A study performed in our lab by Ribeiro et al. determined that HE4 overexpression promotes collateral chemoresistance to both cisplatin and paclitaxel in SKOV3 and OVCAR8 cells (18). Conversely, CRISPR/Cas9 mediated knockdown of HE4 in SKOV3 cells overexpressing HE4 partially reversed their chemoresistance. Microarray analysis revealed suppression of cisplatin-induced early growth response 1 (*EGR1*) gene expression in HE4-overexpressing SKOV3 cells compared with null vector-transfected cells (18). *EGR1* is a transcription factor that regulates apoptosis, proliferation, and differentiation through regulating expression of genes such as p53, BCL2, PTEN, IGF2, PDGF, VEGF, TGFB1, and TNF (114, 115). *EGR1* expression is influenced by MAPK signaling, including phospho-ERK and phospho-p38 (115). Ribeiro et al. found that p38 was strongly activated in SKOV3 null vector-transfected cells treated with cisplatin, while its activation was suppressed in HE4-overexpressing clones (18), suggesting that HE4-mediated chemoresistance may involve MAPK signaling.

Similarly, a study by Wang et al. showed that HE4 represses carboplatin-induced apoptosis *in vitro*. Recombinant HE4 caused an increase in expression of antiapoptotic protein B-cell lymphoma 2 (BCL-2) and a decrease in expression of pro-apoptotic Bax (Bcl-2 associated X protein) in SKOV3 cells treated with carboplatin (9). This decrease in the Bax/Bcl-2 ratio, in addition to the suppression of EGR1 when HE4 is overexpressed, may contribute to the overall decrease in pro-apoptotic factors that leads to chemoresistance in EOC.

#### Microtubule Stabilization

Microtubule-associated protein tau, which has been associated with paclitaxel resistance in ovarian (116), breast (117), and gastric cancer (118), was upregulated in SKOV3 cells overexpressing HE4 compared with null-vector cells (18). In addition, HE4-overexpressing cells were found to express significantly higher levels of SEPT3 (Septin 3) mRNA compared with null-vector controls (18). Septins are a family of conserved GTP binding proteins that are associated with microtubules and actin filaments and have an important role in cytoskeletal organization (119). Furthermore, recombinant HE4 treatment of SKOV3 cells increased β-tubulin levels, indicating that HE4 might promote microtubule stability, leading to paclitaxel resistance.

#### Kinase Signaling Pathways

Human epididymis protein 4 knockdown has also been shown to lead to a reduction in cell growth and the resensitization of ovarian cancer cells to both cisplatin and paclitaxel (12). Lee et al. found that this effect was due to corresponding decreases of ERK and AKT in HE4 knockouts. Activation of these pathways suppresses apoptotic signaling in tumors, suggesting that HE4’s regulation of these pathways may be an important mechanism of chemoresistance (120).

## Steroid Biosynthesis

Evidence suggests an association between sex steroids and EOC pathogenesis, which is explained by processes that take place during the menstrual cycle. The ovarian surface epithelium (OSE) plays a critical role in ovulation and postovulatory wound repair. During the menstrual cycle, the OSE proliferates during the pro-estrus/estrus transition. After, ovulation the proliferation rate decreases (121). It is hypothesized that when the OSE is repeatedly exposed to high doses of luteinizing hormone and follicle stimulating hormone during the menstrual cycle, this can promote cell proliferation and increase the likelihood of tumor growth over time (121). Furthermore, epidemiological data have suggested that ovarian cancer progression, pathogenesis, and etiology are highly dependent on the activity of estrogens (121), and numerous experimental studies have demonstrated the promotive effect of estrogens on ovarian tumors in mice and human EOC cell lines (122). However, activation of diverse oncogenic pathways in EOC may lead to the eventual downregulation of ERα levels and the overall decrease in ERα related signaling in ovarian cancers, rendering them resistant to anti-estrogen therapies (122). Some evidence exists that HE4 may be involved in this process by regulating steroid signaling in EOC. A full list of factors associated with HE4-mediated steroid biosynthesis can be found in Table 1D and is outlined in detail below.

### Steroid Biosynthesis Gene Expression

Two separate microarray pathway analyses identified steroid biosynthesis as a pathway affected by HE4 (10, 74). Important genes that were differentially expressed between HE4-overexpressing and HE4 knockdown cell lines were Forkhead box protein A2 (FOXA2) (74), squalene monooygenase (SQLE), 7-dehydrocholesterol reductase (DHCR7), 24-dehydrocholesterol (DHCR24), and sterol-4-alpha-carboxylate-3-dehydrogenase (NSDHL) (10). FOXA2, a transcription factor required for normal metabolism (123), promotes cell proliferation, maintains cancer stem cells, and is associated with a higher rate of relapse in triple-negative breast cancer (124).

Another gene differentially regulated by HE4, SQLE, is an enzyme required in the later stages of cholesterol synthesis (125). Out of 22 cancer types, SQLE copy number-driven gene expression was highest in breast, ovarian and colorectal cancer (125). Also affected by HE4 levels was DHCR7, one of the terminal enzymes involved in the production of cholesterol from 7-dehydrocholesterol (7DHC). DHCR7 was found to be an important regulatory determinate between cholesterol and vitamin synthesis, as cholesterol is able to accelerate the proteasomal degradation of DHCR7, which can result in the accumulation of 7DHC and an increased production of vitamin D (126). DHCR24, which was also affected by modulation of HE4 levels, is another enzyme in the cholesterol biosynthesis pathway (127). It interacts physically and functionally with DHCR7 (128) and has a number of different cellular functions including anti-inflammatory and antiapoptotic functions, as well as regulation of oxidative stress and cell differentiation (129). DHCR24 has also been proposed to be involved in tumor progression, as its deregulation has been linked to prostate, ovarian, and urothelial carcinomas (127).

Finally, NSDHL is also involved in cholesterol biosynthesis and produces metabolites that are essential in the conversion of squalene to cholesterol (130). Interestingly, NSLD1 was found to have a role in the control of signaling, vesicular trafficking, and degradation of EGFR and its dimerization partners ERBB2 and ERBB3. A study by Sukhanova et al. showed that NSLD1 knockout *in vivo* leads to a reduction in EGFR activation (131). The results from these microarrays show that modulating HE4 levels results in differential expression of several genes involved in steroid biosynthesis—especially cholesterol—suggesting that HE4 may affect tumor metabolism and ultimately contribute to tumorigenesis.

### Estrogen Signaling

In support of the above described pathway analyses, two other studies have shown that HE4 interacts with steroid signaling, specifically estrogen signaling. Lokich et al. showed that ERα expression was reduced in HE4-overexpressing SKOV3 cells, resulting in increased resistance to tamoxifen and fulvestrant compared with wild-type cells (132). 5-Methylcytosine (5-MC), a methylated form of the DNA base cytosine, is one of the most prominently identified epigenetic modifications, and can cause suppression of ERα gene expression. Deregulation of DNA methylation can result in abnormal gene expression and tumorigenesis (133, 134). Lokich et al. found that 5-MC was readily detected in SKOV3 wild-type and null-vector cells but not in HE4-overexpressing clones, suggesting that HE4 overexpression may have an effect on epigenetic modifications (132). However, methylation of the ERα gene was not specifically examined in this study. It is unclear whether HE4 overexpression would promote increased methylation at the ERα promoter region (even with the presence of global demethylation), which would be expected given the reported suppression of ERα in this study.

Interestingly, Chen et al. reported that when HO8910 ovarian cancer cells were stimulated with estradiol (E2), there was an increase in the expression of HE4 at the mRNA and protein level. This effect was not observed in estrogen-insensitive SKOV3 cells; however, when HE4 was knocked down in SKOV3 cells, their proliferative response to estrogen was restored (135). Collectively with the results shown by Lokich et al, this study suggests that HE4 works to suppress estrogen signaling in ovarian cancer cells, which can contribute to resistance to anti-estrogen therapies. Conversely, it appears that estradiol promotes HE4 expression in estrogen-responsive cells, which could indicate a role for HE4 in the initial tumor promoting effects of estrogen. Further clarification of the effect of HE4 on estrogen signaling may be useful in improving implementation of anti-estrogen based therapies.

## Conclusion

Ovarian cancer is an extremely deadly disease owing to the fact that patients are typically diagnosed at a late stage. Initially, patients respond well to frontline platinum therapy; however, a majority of tumors recur, and the initial chemosensitivity eventually gives way to a broad chemoresistance (136). Available detection methods have improved in recent years with the discovery of HE4 as a diagnostic and prognostic biomarker. However, there has yet to be a breakthrough targeted therapy to combat EOC. While PARP inhibitors are used in the maintenance setting for all patients, this therapy has most significantly benefited BRCA-positive patients, who comprise only 20–25% of patients (137, 138). In addition, inhibitors of immune checkpoints, such as programmed death ligand-1 have demonstrated modest benefit in clinical trials for ovarian cancer (139). Therefore, there is still a crucial need for novel targeted EOC treatments.

Although HE4 is well established as a clinical biomarker for ovarian cancer, it has been largely understudied for its therapeutic targeting potential. However, ongoing research continues to support that HE4 is profoundly involved in the pathogenesis of EOC. The individual studies mentioned in this review provide evidence that HE4 promotes EOC progression through pathways associated with cell proliferation, tumor growth, metastasis, chemoresistance, and steroid biosynthesis. These pathways, along with specific genes that have been shown to be associated with HE4, are summarized in Table 1. This compilation of HE4 regulated factors and pathways will serve as a starting point for scientists to further elucidate specific mechanisms by which HE4 ultimately drives tumorigenesis. In addition, a comprehensive summary of clinical, *in vivo*, and *in vitro* studies related to each facet of EOC progression and HE4 can be seen in Figure 1. This diagram highlights the progress that has been made to establish HE4 as an attractive therapeutic target, while simultaneously denoting areas of research that are still lacking. The results discussed here suggest that inhibition of HE4 *via* a neutralizing antibody or small molecule inhibitor could provide viable treatment options for patients in dire need of more effective therapies.

## Author Contributions

NJ, CC, and JR contributed conceptually to this review. All the authors reviewed and approved final manuscript.

## Conflict of Interest Statement

The authors declare that the research was conducted in the absence of any commercial or financial relationships that could be construed as a potential conflict of interest.
